# Ovine *RAP1GAP* and *rBAT* gene polymorphisms and their association with tail fat deposition in Hu sheep

**DOI:** 10.3389/fvets.2022.974513

**Published:** 2022-08-25

**Authors:** Zongwu Ma, Weimin Wang, Deyin Zhang, Yukun Zhang, Yuan Zhao, Xiaolong Li, Liming Zhao, Changchun Lin, Jianghui Wang, Bubo Zhou, Jiangbo Cheng, Dan Xu, Wenxin Li, Xiaobin Yang, Yongliang Huang, Panpan Cui, Jia Liu, Xiwen Zeng, Rui Zhai, Xiaoxue Zhang

**Affiliations:** ^1^College of Animal Science and Technology, Gansu Agricultural University, Lanzhou, China; ^2^The State Key Laboratory of Grassland Agro-Ecosystems, College of Pastoral Agriculture Science and Technology, Lanzhou University, Lanzhou, China

**Keywords:** *RAP1GAP*, *rBAT*, single nucleotide polymorphisms, tail fat deposition, Hu sheep

## Abstract

Excessive fat deposition in the tail of sheep will affect its feed efficiency, which will increase the feeding cost. The purpose of this study was to identify the single nucleotide polymorphisms (SNPs) of *RAP1GAP* and *rBAT* genes by PCR amplification and Sanger sequencing, the SNPs were genotyped by KASP genotyping assays to evaluate their association with tail fat deposition traits. The results showed that two intronic mutations of g.13561 G > A and g.1460 T > C were found in *RAP1GAP* and *rBAT*, respectively. There were three genotypes of GG, AG, AA and CC, CT and TT at these two loci, respectively. Association analysis showed that g.13561 G > A of *RAP1GAP* was associated with tail width, tail fat weight and relative tail fat weight (*P* < 0.05). The g.1460 T > C of *rBAT* was associated with tail width and tail fat weight (*P* < 0.05). Different combinations of genotypes also differed significantly with tail fat deposition traits. In the tail fat tissue, the expression levels of *RAP1GAP* gene was significantly higher in small-tailed sheep than in big-tailed sheep, and the expression levels of *rBAT* gene was significantly higher in big-tailed sheep than in small-tailed sheep. In the liver, the expression levels of *RAP1GAP* and *rBAT* gene was significantly higher at 6 months than at 0 and 3 months. In conclusion, *RAP1GAP* and *rBAT* polymorphisms can be used as a candidate molecular marker to reduce tail fat deposition in sheep.

## Introduction

As a common domestic animal, sheep (*Ovis aries*) are important means of production and livelihood in farming areas, and their meat and wool products play a pivotal role in improving and enhancing local production and livelihood. According to historical records, the wild ancestors of sheep were thin-tailed. Later, the fat-tailed or fat-rumped sheep were bred by artificial selection (1). Tail fat of sheep provides enough energy to maintain normal needs in winter with a shortage of forage (2, 3). With the commercial farming of sheep, the excessive deposition of tail fat limits the commercial value of lamb. It also reduces the feed conversion rate and increases feeding costs. In actual production, it has been found that the mating conception rate of large-tailed sheep is lower than that of small-tailed sheep, because the fat tail hinders natural mating.

Ras-related regulatory protein 1 GTPase-activating protein (*RAP1GAP*) is a protein coding gene, an important regulator of small G protein Rap1 activation, and the first identified member of the family of GTPase-activating proteins (GAPs) (4, 5). It can inhibit extracellular signal-regulated kinase activity, cell proliferation, survival and migration of melanoma cells (6). Leptin is a crucial hormonal mediator to maintain normal weight. *RAP1GAP* is a specific negative regulator of Rap1. The activation of Rap1 will inhibit the cellular effect of leptin and promote fat production (7, 8). Studies have shown that Rap1 gene deletion in the forebrain can protect mice from neuroleptin resistance, obesity and glucose imbalance induced by HFD (9). The *rBAT* (related to b^0, +^ amino acid transporter; SLC3A1) belong to the solute carrier family 3, have been identified (10). The *rBAT* encodes a type II membrane glycoprotein, which is a component of the renal amino acid transport protein that transports neutral and basic amino acids in the renal tubules and intestine. The *rBAT* protein is highly expressed in the apical membrane of epithelial cells of kidney and small intestine (11, 12), forming a heterodimer with b^0, +^ AT (SLC7A9) and AGT1 (SLC7A13) (13, 14). Liver is the hub of energy metabolism in animals. It plays an important physiological role in regulating blood lipid level and cholesterol homeostasis. The liver stores glucose through gluconeogenesis and stores excess fat in adipose tissue. In the study of chickens (*Pullus*), it has been introduced that the liver mediates *de novo* lipogenesis (15). In pigs (*Sus scrofa*), liver and adipose tissue are also the main parts of adipogenesis (16, 17).

As a short fat-tailed breed native to China, Hu sheep is widely welcomed by farmers because of its high reproductive performance, fast growth, strong adaptability, early sexual maturity, annual estrus, large number of lambs and strong lactation ability (18, 19). The *RAP1GAP* and *rBAT* genes have been studied in human medicine, but it is not clear whether they are associated with tail fat deposition in Hu sheep. Therefore, we have two objectives in this study: (1) to explore the relationship between genetic variation of *RAP1GAP* and *rBAT* genes and tail fat deposition traits in sheep. (2) to detect the expression features of *RAP1GAP* and *rBAT* genes in Hu sheep at smallest and largest tail fat weight and at different developmental stages in liver. This study may provide an important candidate molecular markers and theoretical basis for the breeding of thin-tailed fat sheep.

## Materials and methods

### Statement of ethics

All experimental procedures in this study were approved by the Animal Care and Use Committee of Biological Research of Gansu Province, China. And the experimental protocol and sample collection were approved by the Ethics Committee of Gansu Agricultural University (permit number for conducting animal experiments: No. 2012–2-159).

### Experimental animals and extraction of DNA

A total of 1,662 male Hu sheep were used in this study. All lambs purchased from five different companies (including Jinchang Zhongtian Sheep Industry Co. Ltd., Gansu Zhongsheng Huamei Sheep Industry Development Co. Ltd., Gansu Sanyangjinyuan Husbandry Co. Ltd., Shandong Runlin Sheep Industry Co. Ltd., and Wuwei Pukang Sheep Industry Co. Ltd). All lambs were weaned at 56 days of age and immunized before weaning. After weaning, they were transferred to Minqin Defu agricultural Co., Ltd. (Gansu Province, China) for separate feeding. The adaptation period was 14 days, the pre-test period was 10 days and the experimental period was 100 days. During the experiment, maintain the same feeding and environmental conditions. All lambs were raised in a single pen and were allowed to eat and drink freely. Before the end of the experiment, the experimental animals were fed with pellet feed purchased from Gansu Sanyang Jinyuan animal husbandry Co. Ltd. At 180 days, the body weight (BW) was measured with a calibrated electronic scale and tail length and width were measured by the same person using a clearly graduated leather ruler. Carcass and tail fat were weighed after slaughter and a small part of tail fat and other tissues were collected for use in subsequent experiments. We calculate the relative weight of tail fat according to the following formula: the relative weight of tail fat (body weight) = the weight of tail fat / BW, the relative weight of tail fat (Carcass) = the weight of tail fat / carcass weight. Venous blood samples (5 ml) were collected from 1,662 adult sheep and placed in heparin sodium vacuum tubes. Before DNA extraction, they were stored at −20°C. Blood DNA was extracted by EasyPure Blood Genomic DNA kit (TransGen Biotech, Beijing, China), and the DNA of 1,372 sheep was successfully extracted.

### SNP identification and genotyping

The *RAP1GAP* and *rBAT* genes (GenBank Accession No. NC_040253.1 and NC_040254.1) was amplified by PCR. And PCR primers for the *RAP1GAP* and *rBAT* genes were designed according to the DNA base sequence (Table 1). The DNA of 10 Hu sheep was randomly selected as the template DNA for PCR amplification. The PCR reaction system included 17.5 μL of 2 × TSINGKE Master Mix (TSINGKE Biological Technology, Beijing, China), 1.12 μL of forward and reverse primers each, 1.4 μL of sheep template DNA and 14 μL of ddH_2_O. Amplification was performed according to the following procedure: 3 min at 94°C; followed by 30 s at 94°C, 30 s at 45–65°C, and 30 s at 72°C for 35 cycles; final 72°C for 5 min. SNPs in *RAP1GAP* and *rBAT* genes were identified by sequencing of PCR products. All individuals in the study were genotyped with the competitive allele-specific FRET-based PCR (KASPar) assays (LGC Genomics, Hoddesdon, UK). The primers used for genotyping are listed in Table 2.

**Table 1:** Primer pairs for amplification of the ovine *RAP1GAP* and *rBAT* genes.

**Gene**	**Primer name**	**Primer sequences (5'−3')**	**Annealing temperature (°C)**	**Amplicon length (bp)**
*RAP1GAP*	*RAP1GAP*-F *RAP1GAP*-R	CACTCCTCCCACCATCCGTTC CCAGCCTCTCTCCTAGAAACC	58.5°C	937 bp
*rBAT*	*rBAT-F* *rBAT-R*	AATGTGTCCTTCTTCTGTGC GCCACCTTTTAGAATGCTGGA	55.5°C	853 bp
*RAP1GAP*	*RAP1GAP-*expression-F *RAP1GAP-*expression-R	ATTGAAGGCACCAATCACGA CTTGCCGAGAAAGTGCTTCC	63°C	126 bp
*rBAT*	*rBAT-*expression-F *rBAT-*expression-R	GCAGCCATACATGATAAAGGT CTCATCAAAGTGCCAACTGGA	63°C	213 bp

**Table 2:** KASPar genotyping primer.

**Gene**	**Primer**	**Primer sequence (5'−3')**
*RAP1GAP*	Primer_AlleleX	GAAGGTGACCAAGTTCATGCTGAATTTCATAGGTTCAAAGCATCCTTC
	Primer_AlleleY	GAAGGTCGGAGTCAACGGATTAGAATTTCATAGGTTCAAAGCATCCTTT
	Primer_Common	TGGGTGCAGCTTGGCTAGAAGG
*rBAT*	Primer_AlleleX	GAAGGTGACCAAGTTCATGCTGTATAGCTCTAGACGCGGATAC
	Primer_AlleleY	GAAGGTCGGAGTCAACGGATTGGTATAGCTCTAGACGCGGATAT
	Primer_Common	ACTAGCCAAAACCAAACAATTCAGCAAG

### Expression studies of *RAP1GAP* and *rBAT* genes in sheep tail fat and liver tissue

In order to explore the mRNA expression patterns of sheep *RAP1GAP* and *rBAT* genes in tail fat tissue. After slaughter, 12 lambs were selected from the same batch of sheep. According to the characteristics of tail fat deposition (tail width, tail fat weight and relative tail fat weight), they were divided into big-tail group (*n* = 6) and small-tail group (*n* = 6). Supplementary Table 1 shows individual information. Secondly, to explore the mRNA expression level of sheep *RAP1GAP* and *rBAT* genes in liver, the liver tissues of lambs at three different development stages (0, 3, and 6 months of age) were collected, three in each stage. Total RNA was extracted with transzol (TransGen Biotech, Beijing, China), followed by the detection of RNA concentration and purity. RNA extraction was performed by using a reverse transcriptase kit (Takara, Dalian, China), followed by reverse transcription to generate cDNA. The sample preparation and experimental program setup for Quantitative real-time PCR is based on our previous studies (20). Each sample was subjected to four technical repetitions. Data were analyzed using the 2^−Δ*ΔCT*^ method (21), and average relative expression levels of genes were calculated.

### Statistical analysis

According to a previously published method (22), genetic indices such as genotype frequency and allele frequency were calculated based on genotyping results. The association analysis between genotypes and phenotype values based on a linear model in SPSS 25.0 software:


(1) $$\begin{array}{r} Y_{ijkln}=\mu +G_{i}+F_{j}+S_{k}+F_{l}+M_{n}+\varepsilon_{ijkln} \end{array}$$

Where Y_*ijkln*_ is a vector of phenotypic observation, μ is a vector of mean, G_*i*_ represents the effect of the i^th^ genotype, F_*j*_ stands for the effect of *j*^th^ farm (j = 1, 2, 3, 4, 5, 6, 7), S_*k*_ represents the effect of season (k = winter, summer), F_*l*_ and M_*n*_ represent the family effect, and ε_*ijkln*_ was a vector of random residuals. Correlation among genotypic and phenotypic was tested using Duncan test and Tukey test.

## Results

### SNP scanning of sheep *RAP1GAP* and *rBAT* genes

**Figure 1:**
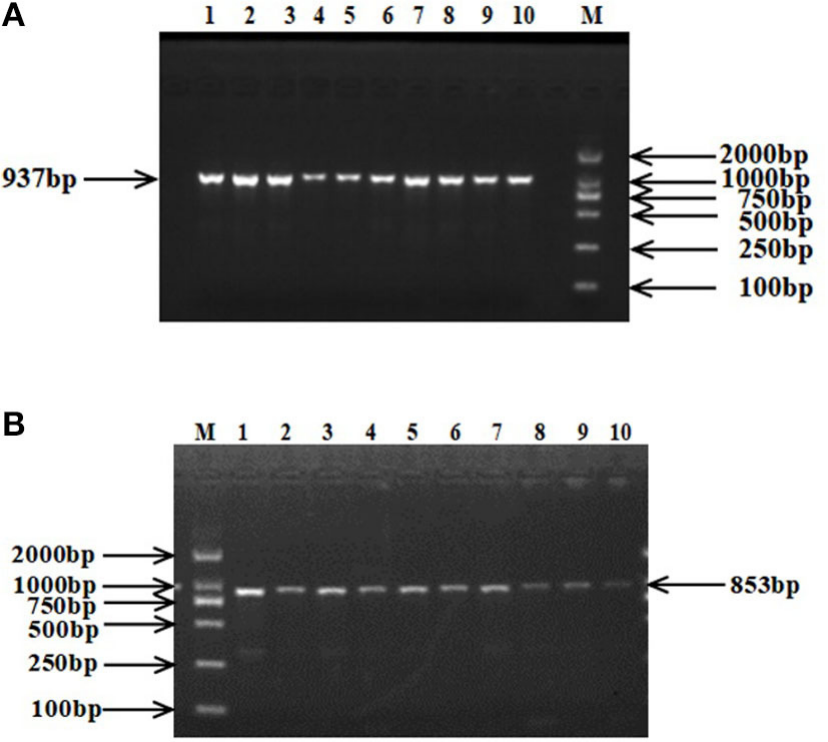
PCR amplification fragments of *RAP1GAP* **(A)** and *rBAT* **(B)** genes. M: DL2000 DNA Marker; 1–10: PCR products.

**Figure 2:**
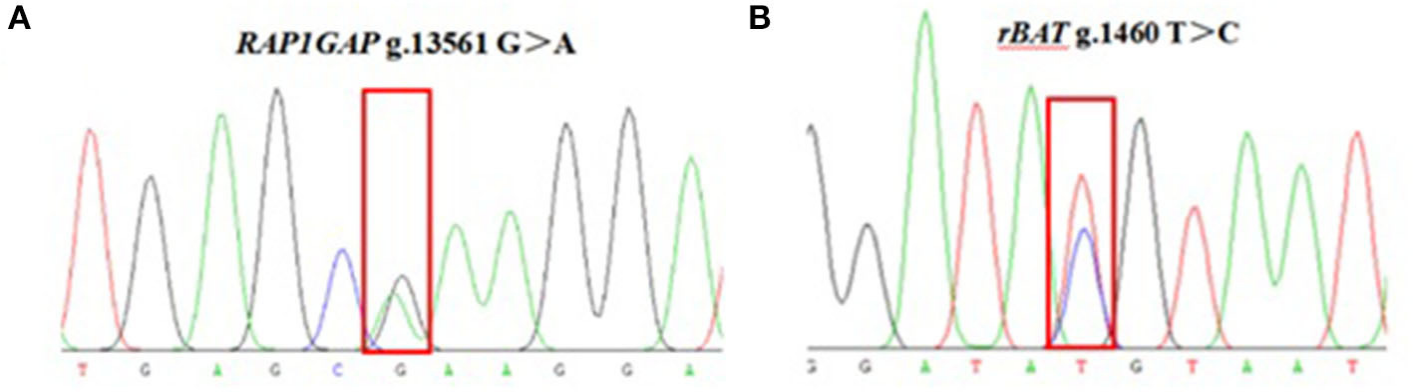
Sequencing peaks of *RAP1GAP* **(A)** and *rBAT* **(B)** loci.

The 937 bp target fragment of *RAP1GAP* gene and 853 bp target fragment of *rBAT* gene were successfully amplified by previously designed primers (Figure 1). Two mutations were discovered within the *RAP1GAP* and *rBAT* genes after sequencing of PCR products (Figure 2). They are intronic located in intron 1 (g.13561 G > A) and intron 1 (g.1460 T > C), respectively.

### Genotyping and genotype and allele frequency analysis

**Figure 3:**
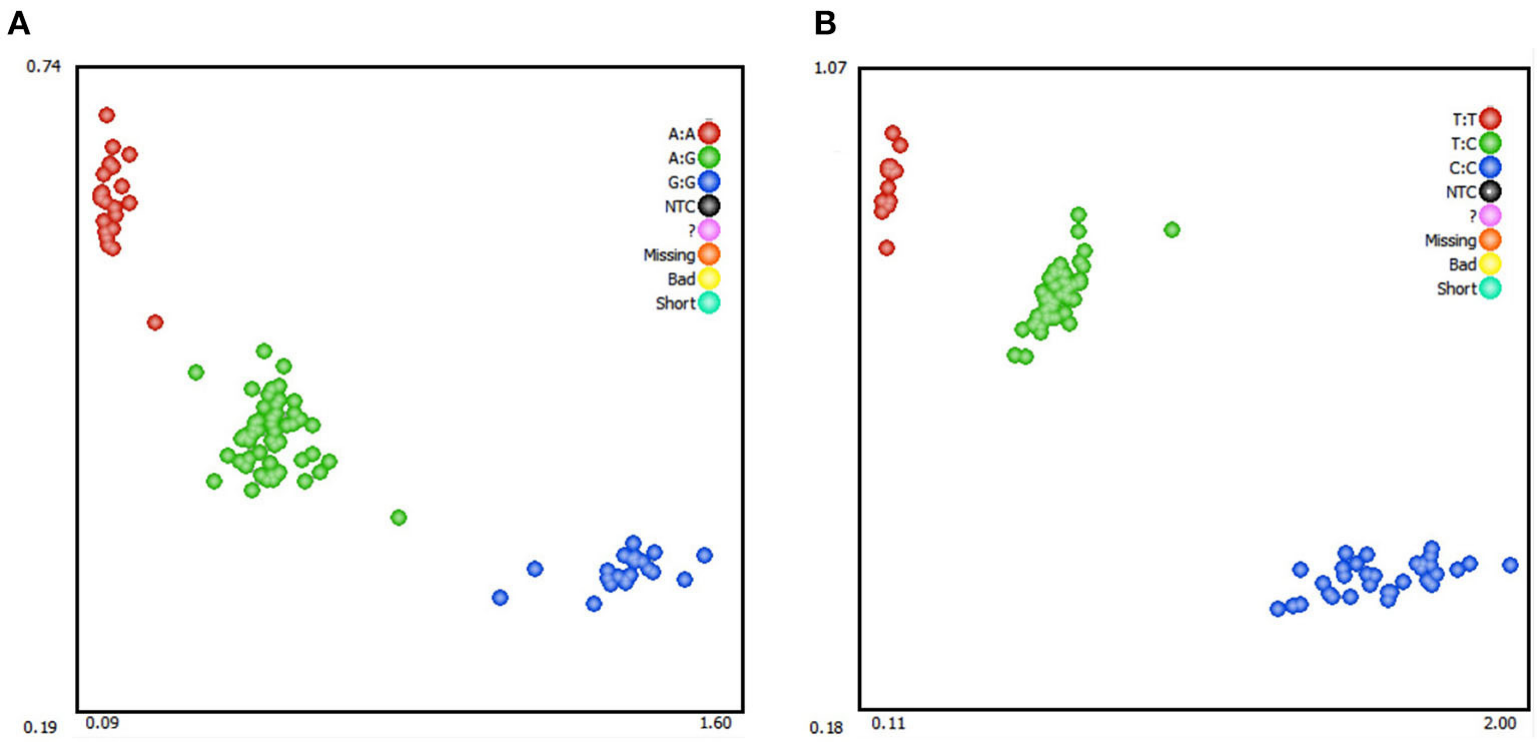
Kaspar-based single nucleotide polymorphism (SNP) genotyping of sheep *RAP1GAP* g.13561 G > A **(A)** and *rBAT* g.1460 T > C **(B)**.

Genotyping of SNPs in *RAP1GAP* and *rBAT* genes by KASPar assays using blood genomic DNA of Hu sheep as template. Three genotypes were detected in both genes: GG, AG, AA (*RAP1GAP*) and CC, CT, and TT (*rBAT*; Figure 3). Within the *RAP1GAP* g.13561 G > A locus, the genotype frequencies of GG, AG, and AA were 0.21, 0.51, and 0.28, respectively. Analysis of genotypic frequencies indicated that A was the dominant allele (0.54). In the *rBAT* g.1460 T > C locus, the genotype frequencies of CC, CT and TT were 0.31, 0.50, and 0.19, respectively. And the C allele was the dominant allele (0.56; Table 3). The PIC, Ne, Ho and He for the *RAP1GAP* gene were 0.38, 2, 0.50, and 0.50, and for the *rBAT* gene were 0.37, 1.96, 0.51, and 0.49, respectively.

**Table 3:** Genotype frequency, allele frequency and genetic diversity at the *RAP1GAP* g.13561 G > A and *rBAT* g.1460 T > C loci.

**Loci**	**Genotype**	**No**.	**Genotype frequency**	**Allele**	**Allele frequency**	**Ne**	**Ho**	**He**	**PIC**
*RAP1GAP* g.13561 G > A	GG	218	0.21	G	0.46	2	0.50	0.50	0.38
	AG	521	0.51						
	AA	290	0.28	A	0.54				
*rBAT* g.1460 T > C	CC	401	0.31	C	0.56	1.96	0.51	0.49	0.37
	CT	643	0.50						
	TT	242	0.19	T	0.44				

### Association analysis of tail fat deposition traits with sheep *RAP1GAP* and *rBAT* genes

The phenotypic distribution of tail weight or relative tail weight in Supplementary Table 2. The association analysis between genotype and phenotype showed that SNPs at the *RAP1GAP* g.13561 G > A and *rBAT* g.1460 T > C locus were significantly associated with the traits of tail fat deposition (Table 4). Specifically, in the *RAP1GAP* gene, the tail width, relative tail fat weight (body weight), and relative tail fat weight (carcass) of individuals with AA genotype was significantly lower than with AG and GG genotypes, and the tail fat weight of individuals with AA genotype was significantly lower than with GG genotype (*P* < 0.05). In the *rBAT* gene, the tail width of individuals with TT genotype was significantly lower than with CT genotype, and the tail fat weight of individuals with TT genotype was significantly lower than with CC genotype (*P* < 0.05). However, the SNP at the *RAP1GAP* g.13561 G > A locus was no significantly associated with 180 day BW and carcass weight (*P* > 0.05). And the SNP at the *rBAT* g.1460 T > C locus was significantly associated with 180 days BW and carcass weight (*P* < 0.05). In a word, the AA and TT genotypes of *RAP1GAP* g.13561 G > A and *rBAT* g. 1460 T > C was the dominant genotypes associated with tail fat deposition in sheep, respectively.

**Table 4:** Association analysis of SNPs in the *RAP1GAP* and *rBAT* genes.

**Gene/loci**	**Item No**.	**BW (kg)**	**Carcass weight (kg)**	**Tail width (cm)**	**The weight of tail fat (kg)**	**The relative weight of tail fat (body weight)**	**The relative weight of tail fat (carcass)**
	GG 218	46.272 ± 0.418	25.623 ± 0.254	17.936 ± 0.162^a^	1.531 ± 0.031^a^	0.032 ± 0.001^a^	0.06 ± 0.001^a^
*RAP1GAP* g.13561 G > A	AG 521	46.111 ± 0.270	25.526 ± 0.164	17.871 ± 0.105^a^	1.512 ± 0.020^ab^	0.032 ± 0.000^a^	0.059 ± 0.001^a^
	AA 290	45.887 ± 0.362	25.387 ± 0.22	17.373 ± 0.141^b^	1.449 ± 0.027^b^	0.031 ± 0.000^b^	0.057 ± 0.001^b^
	GG-AG	0.746	0.750	0.738	0.603	0.586	0.564
*P*-value	GG-AA	0.486	0.484	0.009	0.045	0.025	0.024
	AG-AA	0.620	0.614	0.005	0.060	0.033	0.034
	CC 401	47.049 ± 0.309^a^	26.103 ± 0.188^a^	17.642 ± 0.119^ab^	1.544 ± 0.023^a^	0.032 ± 0.000	0.059 ± 0.001
*rBAT* g.1460 T > C	CT 643	46.267 ± 0.244^b^	25.591 ± 0.148^b^	17.834 ± 0.094^a^	1.504 ± 0.018^ab^	0.032 ± 0.000	0.058 ± 0.001
	TT 242	45.776 ± 0.398^b^	25.169 ± 0.242^b^	17.43 ± 0.154^b^	1.467 ± 0.030^b^	0.031 ± 0.001	0.058 ± 0.001
	CC-CT	0.048	0.033	0.206	0.180	0.419	0.471
*P*-value	CC-TT	0.012	0.002	0.276	0.042	0.232	0.354
	CT-TT	0.293	0.137	0.025	0.288	0.542	0.695

*SNP, single nucleotide polymorphism. The results of association analysis are shown as mean ± standard error. Same column with the same shoulder label or no letter means the difference is not significant (P > 0.05), lowercase letters in different shoulder labels indicate significant differences (P <0.05), or extremely significant differences (P <0.01)*.

### Analysis of *RAP1GAP* and *rBAT* genotypes combinations

As shown in Table 5, by combining the genotypes of the two SNPs loci (*RAP1GAP* g.13561 G > A and *rBAT* g.1460 T > C), we analyzed the effect of different combinations on tail fat deposition traits. The tail width of AA^*RAP*1*GAP*^-TT^*rBAT*^ genotype individuals was significantly lower than AA^*RAP*1*GAP*^-CT^*rBAT*^, AA^*RAP*1*GAP*^-CC^*rBAT*^, AG^*RAP*1*GAP*^-TT^*rBAT*^, AG^*RAP*1*GAP*^-CT^*rBAT*^, and AG^*RAP*1*GAP*^-CC^*rBAT*^ genotypes individuals (*P* < 0.05). The weight of tail fat and the relative weight of tail fat (body weight) of individuals with AA^*RAP*1*GAP*^-TT^*rBAT*^ genotype was significantly lower compared with those of individuals with AG^*RAP*1*GAP*^-TT^*rBAT*^, AG^*RAP*1*GAP*^-CT^*rBAT*^, AG^*RAP*1*GAP*^-CC^*rBAT*^, GG^*RAP*1*GAP*^-TT^*rBAT*^, GG^*RAP*1*GAP*^-CT^*rBAT*^, and GG^*RAP*1*GAP*^-CC^*rBAT*^ genotypes (*P* < 0.05). The relative weight of tail fat (carcass) of AA^*RAP*1*GAP*^-TT^*rBAT*^ genotype individuals was significantly lower than GG^*RAP*1*GAP*^-TT^*rBAT*^ and GG^*RAP*1*GAP*^-CC^*rBAT*^ genotypes individuals (*P* < 0.05). At the same time, combinations between genotypes have an effect on carcass weight and body weight (BW) at 180 days.

**Table 5:** Analysis of combinations between different genotypes of *RAP1GAP* and *rBAT*.

	**Item**	**No**.	**BW (kg)**	**Carcass weight (kg)**	**Tail width 180 (cm)**	**The weight of tail fat (kg)**	**The relative weight of tail fat (body weight)**	**The relative weight of tail fat (carcass)**
	AA*^*RAP*1*GAP*^*-TT*^*rBAT*^*	56	44.170 ± 0.821^c^	24.304 ± 0.499^c^	16.643 ± 0.320^b^	1.344 ± 0.061^b^	0.029 ± 0.001^b^	0.055 ± 0.002^b^
	AA*^*RAP*1*GAP*^*-CT*^*rBAT*^*	140	45.396 ± 0.519^bc^	25.148 ± 0.316^bc^	17.659 ± 0.203^a^	1.468 ± 0.039^ab^	0.031 ± 0.001^ab^	0.058 ± 0.001^ab^
	AA*^*RAP*1*GAP*^**-***CC*^*rBAT*^*	83	47.542 ± 0.674^a^	26.39 ± 0.410^a^	17.578 ± 0.263^a^	1.499 ± 0.050^a^	0.031 ± 0.001^ab^	0.056 ± 0.002^ab^
	AG*^*RAP*1*GAP*^*-TT*^*rBAT*^*	100	46.150 ± 0.614^abc^	25.458 ± 0.374^abc^	17.75 ± 0.240^a^	1.515 ± 0.046^a^	0.032 ± 0.001^a^	0.059 ± 0.001^ab^
Genotype	AG*^*RAP*1*GAP*^*-CT*^*rBAT*^*	243	45.904 ± 0.394^bc^	25.465 ± 0.240^ab^	17.977 ± 0.154^a^	1.511 ± 0.029^a^	0.032 ± 0.001^a^	0.059 ± 0.001^ab^
	AG*^*RAP*1*GAP*^*-CC*^*rBAT*^*	153	46.485 ± 0.497^ab^	25.848 ± 0.302^ab^	17.855 ± 0.194^a^	1.53 ± 0.037^a^	0.032 ± 0.001^a^	0.059 ± 0.001^ab^
	GG*^*RAP*1*GAP*^*-TT*^*rBAT*^*	35	45.912 ± 1.038^abc^	25.086 ± 0.631^bc^	18.057 ± 0.405^ab^	1.523 ± 0.077^a^	0.033 ± 0.001^a^	0.061 ± 0.002^a^
	GG*^*RAP*1*GAP*^*-CT*^*rBAT*^*	116	46.237 ± 0.570^ab^	25.648 ± 0.347^ab^	18.091 ± 0.223^ab^	1.505 ± 0.042^a^	0.032 ± 0.001^a^	0.059 ± 0.001^ab^
	GG*^*RAP*1*GAP*^*-CC*^*rBAT*^*	56	46.860 ± 0.821^ab^	25.977 ± 0.499^ab^	17.455 ± 0.320^ab^	1.575 ± 0.061^a^	0.032 ± 0.001^a^	0.060 ± 0.002^a^

*The results of association analysis are shown as mean ± standard error. Same column with the same shoulder label or no letter means the difference is not significant (P > 0.05), lowercase letters in different shoulder labels indicate significant differences (P <0.05), or extremely significant differences (P <0.01)*.

### Group statistics

**Figure 4:**
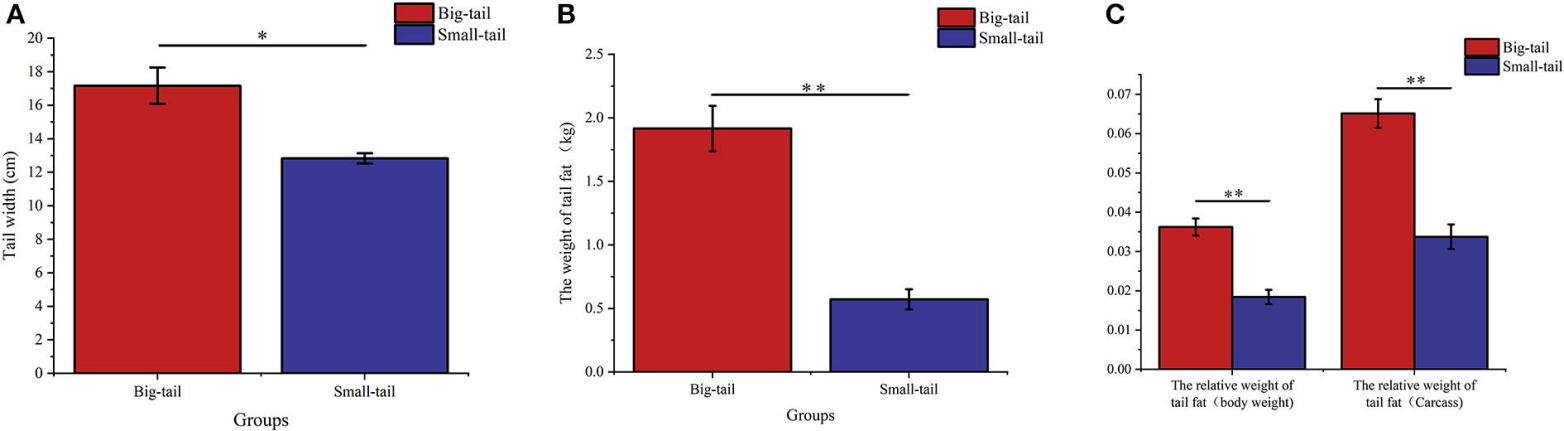
Phenotypic values of tail width **(A)**, tail fat weight **(B)**, and relative tail fat weight **(C)** of grouped individuals, * indicates a significant difference between the big-tail group and the small-tail group (*P* < 0.05), ** indicates a very significant difference between the big-tail group and the small-tail group (*P* < 0.01).

We chose big-tail sheep (*n* = 6) and small-tail sheep (*n* = 6) to study the expression of *RAP1GAP* and *rBAT* genes in tail fat, and calculated the relative weight of tail fat according to the statistics of previous phenotypic data. The tail width, tail fat weight and relative tail fat weight of grouped individuals are shown in Figures 4A–C. The results showed that there was significant difference between the big-tail group and the small-tail group (*P* < 0.05).

### *RAP1GAP* and *rBAT* genes expression in sheep small-tail and big-tail groups

**Figure 5:**
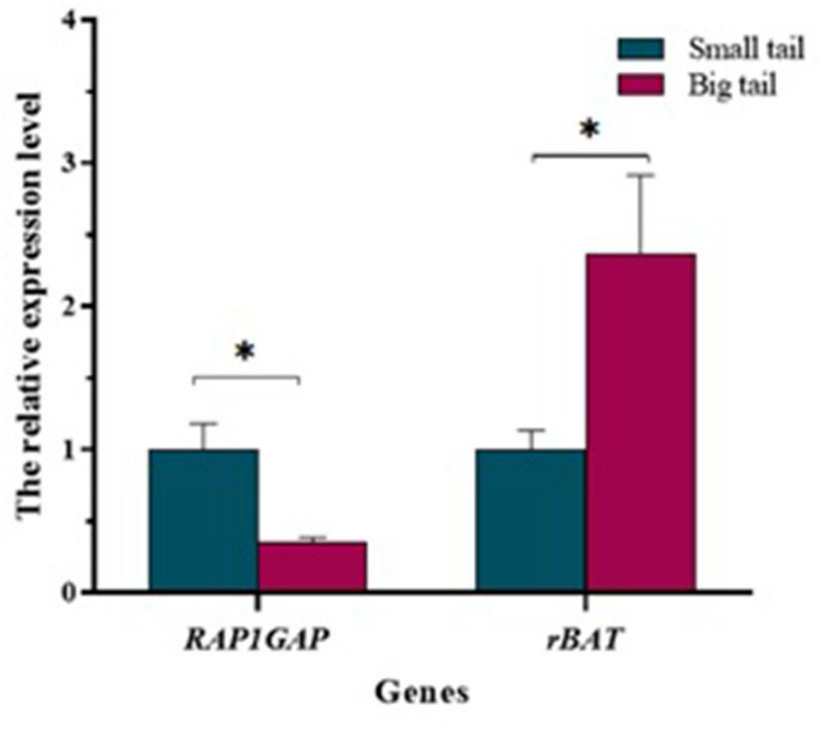
Relative expression of *RAP1GAP* and *rBAT* mRNA between the small-tail and big-tail groups. Asterisks indicate significant differences between the small- and big-tail groups (*P* < 0.05).

In the tail fat tissue, the results of qRT-PCR showed that the level of *RAP1GAP* gene expression was significantly higher in the small-tail sheep group than in the big-tail sheep group (*P* < 0.05). However, *rBAT* gene was significantly lower in the small-tail sheep group than in the big-tail sheep group (*P* < 0.05; Figure 5).

### Expression of *RAP1GAP* and *rBAT* genes in liver at different developmental stages

**Figure 6:**
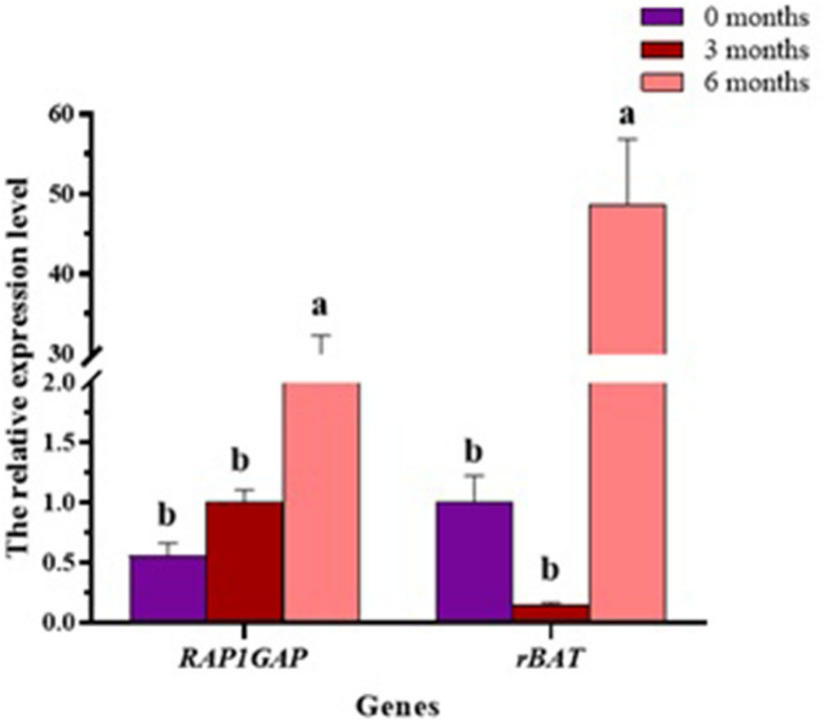
In the liver, the relative expression of *RAP1GAP* and *rBAT* at different developmental stages. The same or no letters indicate a non-significant difference (*P* > 0.05), different letters indicate a significant difference (*P* < 0.05).

To investigate *RAP1GAP* and *rBAT* gene expression in different developmental stages of the liver, we collected liver tissue samples from sheep at different stages of development (0, 3, and 6 months of age), three for each stage. The results show that in liver tissue, *RAP1GAP* and *rBAT* gene expression did not change significantly at 0–3 months of age, but increased dramatically at 6 months of age (*P* < 0.01; Figure 6).

## Discussion

High fat diet is easy to lead to obesity and a series of diseases, which is one of the reasons for the change of people's diet structure. People prefer high protein and low-fat diet. In addition, in the intensive breeding of sheep, tail fat is no longer important in providing energy during the cold season, and the excessive deposition of tail fat increases the breeding cost and reduces the meat quality grade. Therefore, in order to maximize economic benefits and reduce feeding costs, commercial breeding urgently needs to cultivate sheep varieties with small tail shape. Nowadays, the mature application of marker assisted selection (MAS) may make this need a reality.

Adding the same amount of fat and lean tissue to the tail requires a greater energy cost. In addition, the price of fat tail per kilogram is lower than that of red meat. Therefore, sheep producing small fat tail have more economic value in actual production (23). Understanding the genetic mechanism of sheep tail size is very important for future sheep molecular breeding. Previous studies have found that overexpression of *RAP1GAP* has a negative regulatory effect on Rap1, which can enhance the cellular effect of leptin and reduce the production of obesity (6–8). *rBAT* mutation can cause damage to the transport of plasma membrane, and then affect the outflow of arginine in cells (24). Arginine supplementation increases lipolysis and inhibits lipogenesis in white adipose tissue (25). Therefore, it is very important to explore the potential effects of *RAP1GAP* and *rBAT* genes on important economic traits of sheep. Sanger sequencing showed that two novel polymorphic sites (g.13561 G > A and g.1460 T > C) were found in the intron region of *RAP1GAP* and *rBAT* genes in sheep. It is well-known that intronic mutations do not change amino acid sequences. However, relevant studies have shown that mutations located in introns may have similar effects to synonymous mutations (26–28), which can affect some important cellular processes, such as mRNA stability (29), protein folding and function (30), mRNA translation (31), mRNA folding (32), or splicing (33). The occurrence of intronic mutations can affect some important economic traits of animals, such as growth traits (20), tail fat deposition traits (34), feed efficiency (35), and litter size (36). To investigate whether intronic mutations affect phenotypes in sheep, this study assessed the relationship between different genotypes and tail fat deposition traits. The results revealed that the *RAP1GAP* gene with a SNP of g.13561 G > A and the *rBAT* gene with a SNP of g.1460 T > C were significantly associated with tail fat deposition traits (*P* < 0.05). We also found that the combined genotypes of *RAP1GAP* g.13561 G > A and *rBAT* g.1460 T > C also differed for tail fat deposition, which is consistent with previous reports (34).

At the same time, the expression of *RAP1GAP* and *rBAT* genes was detected in adipose tissue of big-tail group and small-tail group. The results showed that the expression of *RAP1GAP* gene was abundant in the small-tail group. This may be because the extensive expression of *RAP1GAP* gene in the small-tail group inhibits Rap1, enhances the cellular effect of leptin, and thus reduces fat deposition (9). It may also be that the extensive expression of *RAP1GAP* gene inhibits the proliferation and survival of tail adipocytes (6). The expression of *rBAT* gene is abundant in the big tail group, which may be because *rBAT* mutation leads to the weakening of plasma membrane transport capacity, affecting the outflow of arginine in cells, and the reduction of arginine promotes the production of fat (24, 25). In addition, the liver plays an important physiological role in fat production (37). In ruminants and humans, the endogenous fat released by fat decomposition increases the content of circulating non-esterified fatty acids into the liver, which is the main source of triglyceride accumulation in the liver (38). It was found that *RAP1GAP* and *rBAT* genes were widely expressed in the liver at the age of 6 months.

In conclusion, *RAP1GAP* and *rBAT* genes might play an important role in the development of adipocytes. Therefore, the polymorphisms of *RAP1GAP* and *rBAT* genes are of great significance for the breeding of smaller tail sheep, and can be used as candidate molecular markers. There are still some limitations in our research. We analyzed the association between polymorphisms of *RAP1GAP* and *rBAT* genes and tail fat deposition traits, which was verified in Hu sheep population. However, further studies are needed to verify its relationship with other breeds of big tailed sheep and its function at the cellular and protein levels.

## Conclusion

In this study, a novel study was performed to explore the presence of polymorphisms on ovine *RAP1GAP* and *rBAT* genes, to investigate their association with tail fat deposition traits in Hu sheep, and to detect their expression features in tail fat and liver tissue. The results showed that two SNP g.13561 G > A and g.1460 T > C were identified in *RAP1GAP* and *rBAT* genes which were significantly associated with tail fat deposition traits. In addition, the expression level of *RAP1GAP* gene in the small-tail group was more abundant than that in the big-tail group, on the contrary, the expression of *rBAT* gene was more abundant in the big-tail group. During the development of liver, the expression levels of *RAP1GAP* and *rBAT* genes peaked at 6-month of age. Therefore, the two newly discovered polymorphic loci can be used as potential molecular markers for breeding smaller tailed sheep.

## Data availability statement

The datasets presented in this study can be found in online repositories. The names of the repository/repositories and accession number(s) can be found below: GenBank Accession Nos. NC_040253.1 and 106 NC_040254.1.

## Ethics statement

The animal study was reviewed and approved by Ethics Committee of Gansu Agricultural University. Written informed consent was obtained from the owners for the participation of their animals in this study.

## Author contributions

XZh, WW, and ZM conceived and designed the study. YZhan, YZhao, JW, BZ, WL, CL, JC, DX, XZe, JL, RZ, YH, and PC collected samples. ZM, LZ, DZ, XL, and XY performed the experiments and analyzed the data. ZM wrote the paper. XZh revised the manuscript. All authors contributed to the article and approved the submitted version.

## Funding

This work was supported by the National Natural Science Foundation of China (31960653), the National Joint Research Program for Animal and Poultry Breeding (19210365), and the Gansu Provincial Key Research and Development Program (20YF3NA012).

## Conflict of interest

The authors declare that the research was conducted in the absence of any commercial or financial relationships that could be construed as a potential conflict of interest.

## Publisher's note

All claims expressed in this article are solely those of the authors and do not necessarily represent those of their affiliated organizations, or those of the publisher, the editors and the reviewers. Any product that may be evaluated in this article, or claim that may be made by its manufacturer, is not guaranteed or endorsed by the publisher.
