# Supplementary material for: Ovine RAP1GAP and rBAT gene polymorphisms and their association with tail fat deposition in Hu sheep
Source: Front Vet Sci. 2022 Aug 25;9:974513. doi: 10.3389/fvets.2022.974513 (PMC9453205; doi:10.3389/fvets.2022.974513)
Supplement: Supplementary file 1 [file Data_Sheet_1.PDF]

Table S1. Descriptive statistics of tail fat related traits.

Items	Mean	SD	CV (%)	Min	Max
Body weight (kg)	46.583	6.451	13.849	24.550	71.950
Carcass weight (kg)	25.556	3.865	15.124	11.600	39.600
Tail width (cm)	17.718	2.401	13.556	10.000	28.000
The weight of tail fat (kg)	1.487	0.475	31.926	0.310	3.715
The relative weight of tail fat（body weight)	0.031	0.008	26.387	0.009	0.074
The relative weight of tail fat（Carcass)	0.058	0.015	25.556	0.018	0.148

Table S2. Descriptive statistics of tail fat deposition in sheep of the big-tail group and small-tail group.

Item	Number	Tail width (cm)	The weight of tail fat (kg)	The relative weight of tail fat (body weight)	The relative weight of tail fat (Carcass)
Big-tail group	BT1	18	1.97	0.04	0.07
	BT2	18	18	0.04	0.07
	BT3	15	1.74	0.03	0.06
	BT4	19	2.06	0.04	0.08
	BT5	13	1.14	0.03	0.05
	BT6	20	2.23	0.04	0.07
Small-tail group	ST1	13	0.82	0.02	0.04
	ST2	12	0.42	0.01	0.02
	ST3	13	0.55	0.02	0.03
	ST4	14	0.79	0.02	0.04
	ST5	13	0.51	0.02	0.03
	ST6	12	0.34	0.02	0.03
